# FT-IR Study, Thermal Analysis, and Evaluation of the Antibacterial Activity of a MK-Geopolymer Mortar Using Glass Waste as Fine Aggregate

**DOI:** 10.3390/polym13172970

**Published:** 2021-08-31

**Authors:** Giovanni Dal Poggetto, Antonio D’Angelo, Ignazio Blanco, Simona Piccolella, Cristina Leonelli, Michelina Catauro

**Affiliations:** 1 Department of Engineering “Enzo Ferrari”, University of Modena and Reggio Emilia, Via P. Vivarelli n. 10, 41125 Modena, Italy; giovanni.dalpoggetto@unimore.it (G.D.P.); cristina.leonelli@unimore.it (C.L.); 2 Department of Environmental, Biological and Pharmaceutical Sciences and Technologies, University of Campania “Luigi Vanvitelli”, Via Vivaldi 43, 81100 Caserta, Italy; antonio.dangelo@unicampania.it (A.D.); simona.piccolella@unicampania.it (S.P.); 3 Department of Engineering, University of Campania “Luigi Vanvitelli”, Via Roma n. 29, 81031 Aversa, Italy; 4 Department of Civil Engineering and Architecture and UdR-Catania Consorzio INSTM, University of Catania, Viale Andrea Doria 6, 95125 Catania, Italy; iblanco@unict.it

**Keywords:** geopolymer, metakaolin, waste glass, FT-IR, thermal analysis, antibacterial properties

## Abstract

Food containers made from glass are separately collected from urban solid waste at 76% in most parts of Europe. The cullet glass finds its way to re-melting, while the debris is often disposed of. With this contribution, we suggest an upcycling process where glass debris is simply ground without any washing operation and added to an alkali-activated paste. Metakaolin-based geopolymer mortar added with coarsely ground glass waste as fine aggregate has been prepared via alkali activation with NaOH and Na-silicate. After 7, 14 and 28 days of room temperature curing time, the 3D geopolymer network was investigated by Fourier-transform infrared spectroscopy (FT-IR). Vibrational spectra revealed the geopolymerization occurrences, results which have been supported by both FT-IR deconvoluted spectra and thermogravimetric analysis (TGA). Finally, the antibacterial properties were investigated against both gram-negative (*E. coli*) and gram-positive (*E. faecalis*) bacterial strains. The results suggest the ability of the 28 days cured geopolymers to inhibit the growth of the gram-negative bacterium assayed.

## 1. Introduction

Within recent decades, geopolymers have become suitable replacements for conventional concrete materials, and many efforts have been carried out to improve their functional properties while, at the same time, trying to increase their environmental performance [1].

The alkali activation of alkali aluminosilicate glass yielded cements with high compressive strength (65 MPa after 20 h at 85 °C) [2], and the alkali activation of vitreous calcium aluminosilicate derived from glass fiber waste generated mortar samples with even higher compressive strengths (up to 77 MPa after 3 days at 65 °C) [3]. Similarly, the alkali activation of container glass, or typical soda-lime glass, already proved to be a convenient upcycle procedure for this common waste. Waste glass has been added as solid [4,5,6,7] to an alkali activated matrix, or it has been directly alkali activated, typically with soda, to substitute the water glass solution as an activator [8,9,10]. In order to increase energy saving, the curing of the alkali activated formulations can occur at room temperature [7]. In this particular condition, the binding efficiency of container glass is limited, making the addition of other binders, such as metakaolin, essential. In a recent paper, Abdollahnejad et al. [11] compared the mechanical properties of waste glass-based geopolymers with and without the addition of lime, revealing that after 28 days of curing time, the formulation obtained with both additives possessed increased compressive strength (10 MPa) with respect to the one without the lime (0.5 MPa). Limiting our investigation to alkali activation, another option for the binder is fly ash. Jiang et al. 2020 [12] reported the influence of heat treatment (from 20 to 1200 °C) on the compressive strength of waste glass added to fly ash-based geopolymers. The authors highlighted that when the heat treatment was lower, the compressive strength was higher. After treating at 20 °C, the geopolymer made up of 20 wt % of waste glass and 80 wt % of fly ash has a compressive strength of ca. 54 MPa, but a heat treatment up to 1200 °C highly decreases the compressive strength (less than 5 MPa). The investigation of the alkali activated matrix, in our case the metakaolin-based geopolymer matrix, with increasing temperature is extremely interesting and not well-explored when waste glass is added. 

Additionally, we should remark that among the techniques employed to study geopolymerization, Fourier-transform infrared spectroscopy (FT-IR) is the one widely used. Many papers [10,13,14,15,16,17] report the shift of the main peaks (also called the density state of peak maximum or DOSPM) to lower wavenumbers after the alkali activation of the precursors. Indeed, these shifts are directly correlated to the polymerization degree of the 3D-network. In addition, there are several papers that focus their attention on deconvoluted DOSPMs to better understand the contributions of Si-O-T (where T= Si or Al) vibrations in synthesized geopolymers [7,18,19,20,21]. Geopolymer thermal properties are another important feature to take into account [22,23,24]. It has been reported that a heat treatment up to 900 °C of metakaolin or fly ash-based geopolymer strengthens its mechanical properties. This occurs by means of shrinkage phenomena due to the water removal and to an increasing of the structure’s compactness [25]. Furthermore, Nergis et al., 2020, compared the thermal analysis of geopolymers based on fly ash, waste glass, and sand. The samples made containing 70% of sand, 15% of fly ash, and 15% of waste glass had a weight loss of 3% up to 800 °C, while the sample obtained only with fly ash and waste glass had a weight loss of 18% up to 800 °C [26].

In a previous paper it was observed that the fine waste glass powders have a good reactivity at room temperature and up to 60 wt % in a metakaolin based geopolymer [7]. Additionally, we proved that the thermal properties and the ecological impact were not depleted by the presence of unwashed waste glass [10]. In this paper, we investigated the addition of unwashed ground waste glass, with a grain size below 150 μm, recovered from beverage containers. The motivation for this particular research work was to combine fine and coarse grain sizes in order to support alkaline reactivity and the densification of the MK with additional reactive aluminosilicate (fine fraction), and to create a filler (coarser fraction) capable of increasing the mechanical performance. At the same time, by planning one single sieving operation, we reduced the processing costs and obtained a mortar with waste glass in place of sand. We characterized the consolidated final materials by carefully evaluating the formation of Al-O-Si bonds with FT-IR spectroscopic investigation and peak elaboration, correlating such results with chemical stability in water and thermal stability up to 1400 °C.

## 2. Materials and Methods

### 2.1. Materials

**Figure 1:**
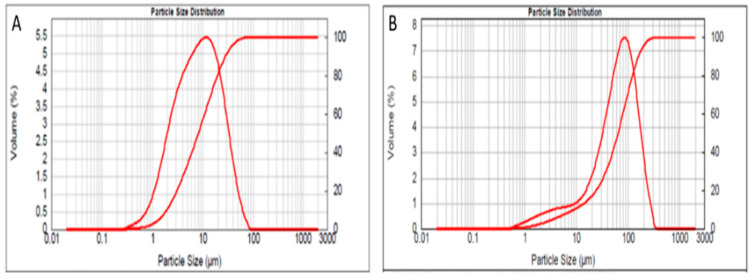
Particle size distribution and cumulative volume for: (**A**) MK and (**B**) WG.

Reactive metakaolin (MK) (Argical™-M 1000, Imerys, France) and waste glass (WG) were used as geopolymer solid aluminosilicate precursors. The MK mineralogical composition was reported elsewhere in a recent work [7], while WG powders were obtained from dry grinding of as-received container glass, with a typical soda-lime composition, and sieved < 160 µm. Figure 1A,B show the particle size distributions of MK and WG powders after grinding, identifying the respective characteristic diameters d (0.1): 1.980 μm, d (0.5): 8.383 μm, d (0.9): 28.793 μm for MK and d (0.1): 9.270 μm, d (0.5): 64.391 μm, d (0.9): 158.958 μm for WG particles. 

Sodium hydroxide (NaOH), sodium silicate (Na_2_SiO_3_) with SiO_2_/Na_2_O = 2.58, acetone (C_3_H_6_O), and KBr of an analytical grade, were purchased from Sigma Aldrich. NaOH pellets were dissolved in MilliQ water obtaining NaOH 8 M used as an alkali solution for activation.

### 2.2. Preparation of Geopolymer Specimens

**Figure 2:**
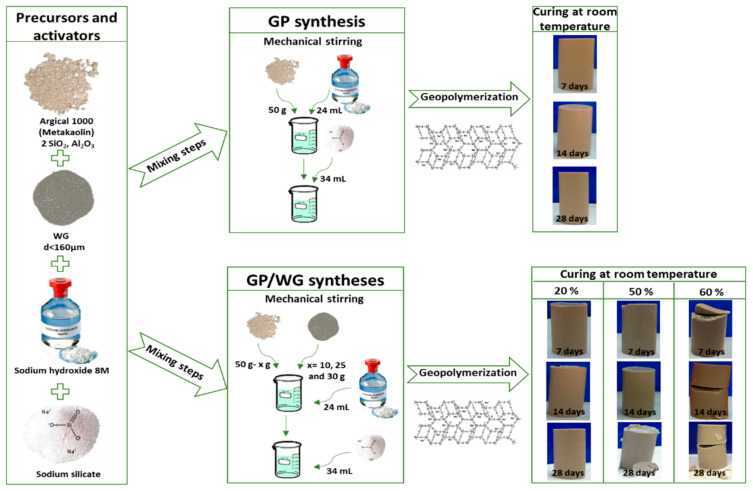
Flow diagram of GP and GP/WG (20, 50 and 60%) formulations and extractions.

The syntheses of sole metakaolin-based geopolymer, hereafter indicated as GP, and its mortars prepared with the addition of 20, 50, and 60 wt % of waste glass, are reported in Figure 2. NaOH 8 M solution was slowly added to MK under mechanical stirring until the mixture became quite solid. Then Na_2_SiO_3_ was added to the mixture and stirred until a homogeneous paste was achieved. The paste was poured into the plastic cylinders that were carefully closed after removing the bubbles. The samples were stored at room temperature at 7, 14, and 28 days of curing time. All the other samples were prepared with the same procedure by varying mass ratio between MK and WG.

### 2.3. Geopolymer Characterizations

#### 2.3.1. Ionic Conductivity and pH Measurements

MilliQ water (1:10 solid-water ratio) was added to the grounded and sifted (d < 125 µm) samples. After shaking the solution, we waited a short time for the solids to sediment prior to our analyses. After waiting for the pH and conductivity stability, the values were collected on samples after 7, 14, 21, and 28 days of curing time, respectively. Ionic conductivity and pH measurements were performed with a Crison GLP31 and Crison GLP21, respectively. All the measurements were made in triplicate to calculate the mean standard deviation.

#### 2.3.2. FT-IR Analysis

FT-IR analysis was performed in the range of 400–4000 cm^−1^ using the Prestige21 Shimadzu system equipped with a DTGS KBr (deuterated triglycine sulfate with potassium bromide windows) detector at a resolution of 2 cm^−1^ (45 scans). The analysis procedure used KBr disks (2 mg of powdered sample mixed with 200 mg of KBr). FT-IR spectra were elaborated by IRsolution and Origin 9 software. The analyses were performed on the samples cured at 7, 14, and 28 days at room temperature. The spectra were deconvoluted in the range of 750–1300 cm^−1^ using a Peak deconvolution tool (from Origin 9) with Gaussian peak shapes and variable peak widths. The fitting process was performed in agreement with Zhang et al. 2012 [18], El-Naggar and El-Dessouky 2017 [19], and Rovnaník 2010 [20], and combined with the software function of self-fitting, minimizing the number of component bands while retaining a regression coefficient R^2^ value above 0.999.

#### 2.3.3. Thermogravimetric Analysis

A thermogravimetric analysis (TGA) of both raw materials (MK and WG) and the geopolymer mortars was carried out using the simultaneous DSC/TGA and discovery SDT650. For this analysis, the samples were grounded to obtain a powder. An amount of 20–30 mg of powders were weighed and placed into an alumina crucible. A nitrogen atmosphere was used with a flow rate of 100 mL/min. The temperature was raised from 20 to 1400 °C under a heating rate of 10 °C min^−1^ as the best resolution rate. The results were produced with the software TRIOS of TA instruments.

#### 2.3.4. Antibacterial Test

**Figure 3:**
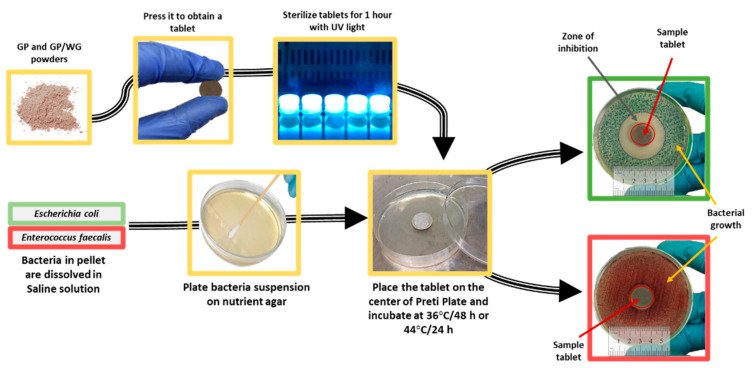
Flow diagram of experimental setup used for the determination of the antibacterial activity of GP and GP/WG (20, 50 and 60%).

The antibacterial test procedure is reported in Figure 3. *Escherichia coli* (ATCC 25922) and *Enterococcus faecalis* (ATCC 29212) were grown in the absence and presence of the synthesized materials (100 mg per tablet) extracted after 28 days at room temperature. Before plating the bacteria, 10^5^ CFU/mL were obtained by diluting the strains in distilled saline water (0.9% NaCl). The diameter of the inhibition halos (IDs), in relation to the Petri Plate diameter (PPD) (6 cm), were measured. Four measurements for each sample were carried out to determine the mean standard deviation. 

## 3. Results and Discussion

### 3.1. Sample Observations

The pictures of the samples extracted at different curing times (Figure 2) show that GP and GP/WG 20% samples had never broken during the demolding procedures, while the samples with the highest content of WG (50 and 60%) had cracked at middle or at lower base. Moreover, we noticed that the GP and GP/WG 20% were completely hardened with respect to the GP/WG 50, and that 60% showed an aliquot of unreacted alkaline solution on the upper surface. The unreacted solution was due either to the impermeability of glass powder, which differs from MK which has no mesopores to collect such solutions, or the need for a more efficient formulation with a reduced alkaline solution. 

### 3.2. pH Measurement

**Figure 4:**
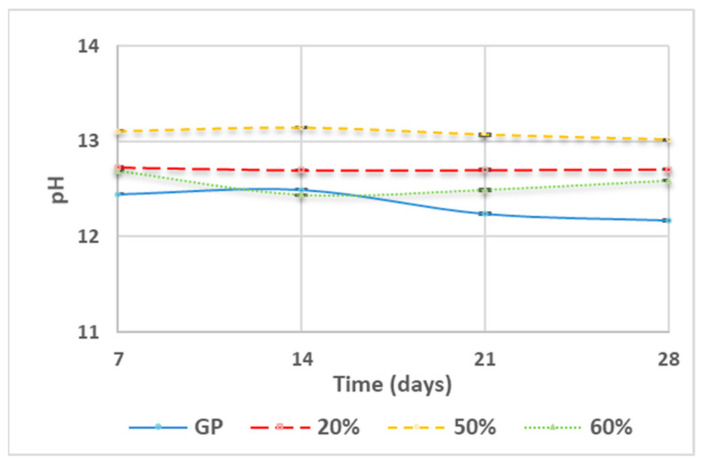
pH of the solution obtained immersing in water the samples of GP and GP/WG (20, 50 and 60%) at different curing times. Error associated with this measurement is approx. 2%.

Figure 4 reports the pH of the solution measured after the immersion of the samples cured at different times. Generally, an increase in WG content corresponds to an increase in the alkalinity of the solutions. GP (curve blue) showed the lower pH value stabilized at 12.2 after 21 and 28 days of curing time. GP/WG 20% (curve red) and 50% (curve yellow) possessed the higher alkaline pH (12.7 and 13.1, respectively) with minimal changes at different curing times. Finally, GP/WG 60% (curve green) had an unstable pH tendency, where at 7 days the pH was 12.7, at 14 days it decreased to 12.4, and after 21 and 28 days it increased up to 12.7.

In conclusion, being that the pH values were gathered in a reduced range, namely from 12 to 13, we deduced that the unreacted alkaline solution had a very similar concentration in all the samples. This means that the first step of the alkali activation mechanism, i.e., the aluminosilicate dissolution, has proceeded in a very similar way for all the compositions and for all the curing times. This conclusion is coherent with one of the two hypotheses reported in point 3.1 to explain the residual solution in the samples with a higher WG content: as for the formulations with higher MK content, the remaining solution was collected at the top surface of the specimen rather than being distributed in the mesopores. 

### 3.3. Ionic Conductivity

**Figure 5:**
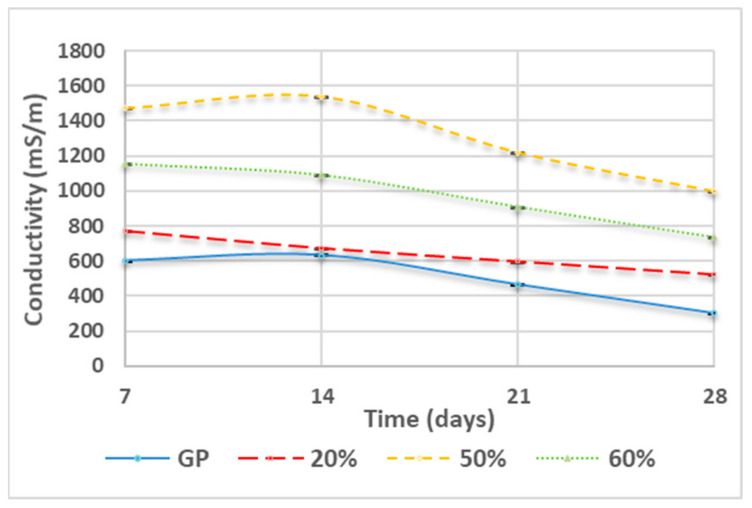
Conductivity of GP and GP/WG (20, 50 and 60%) at different curing times. Error associated with this measurement is approx. 8%.

Figure 5 illustrates the conductivity at different curing times. Generally, an increase in WG content (see curve red, green, and yellow) corresponds to increased conductivity. For example, GP (curve blue) and GP/WG 20% (curve red) showed the lowest conductivity values, while GP/WG 50% (curve yellow) possessed higher conductivity values. From the figure, one can see, more generally, that an increase in waste content is linked to an increase in the conductivity of the solutions and that all these values tend to decrease during the increase of curing time.

### 3.4. FT-IR Spectra

**Figure 6:**
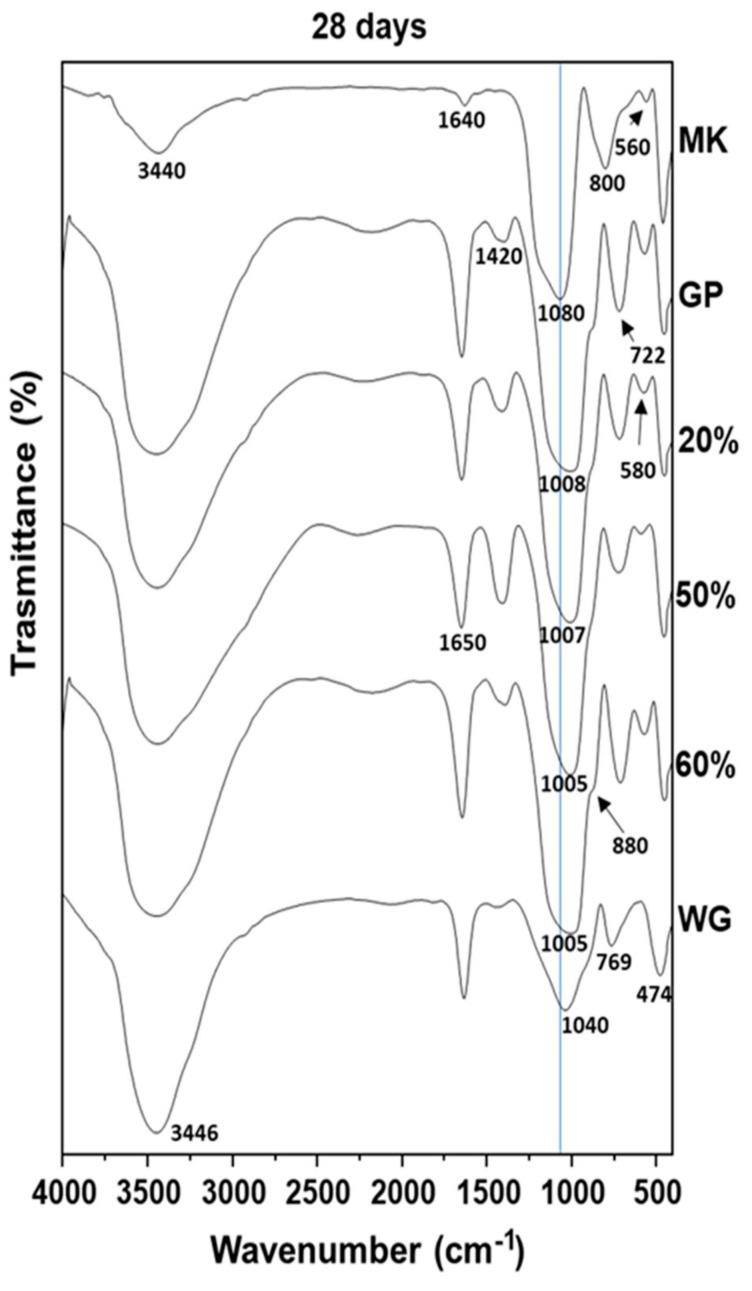
FT-IR comparison spectra of MK, WG, GP and GP/WG samples extracted after 28 days of curing time. The percentages indicate the waste glass content.

Figure 6 reports the FT-IR spectra of MK, WG, GP, and GP/WG (20, 50 and 60%) extracted after 28 days of curing. In all the spectra, the bands at 3440 cm^−1^ and 1650–1640 cm^−1^ are assigned to stretching and bending modes of the -OH groups of water [14,27,28]. The peak detected at 800 cm^−1^, ascribed to Si-O in the MK spectrum, indicates the presence of quartz [14], while the absorption band at 560 cm^−1^ is due to the presence of Al-O vibrations of Al^+6^ [14,29]. Moreover, in GP and GP/WG samples, the absorption band at 1420 cm^−1^ is assigned to carbonate stretching [30], and the bands at 470–450 cm^−1^ are ascribed to the Si-O-Si and O-Si-O bending modes [14,27]. Regarding the absorption bands in the range of 1300–800 cm^−1^ (Si-O asymmetric stretching), reported as DOSPM (density of state of peak maximum) by different authors, there a shift to lower wavenumbers (1008–1005 cm^−1^) with respect to the MK DOSPM (1080 cm^−1^), which suggests an occurrence between the geopolymerization and the formation of the 3D-networks [13,14,17].

**Figure 7:**
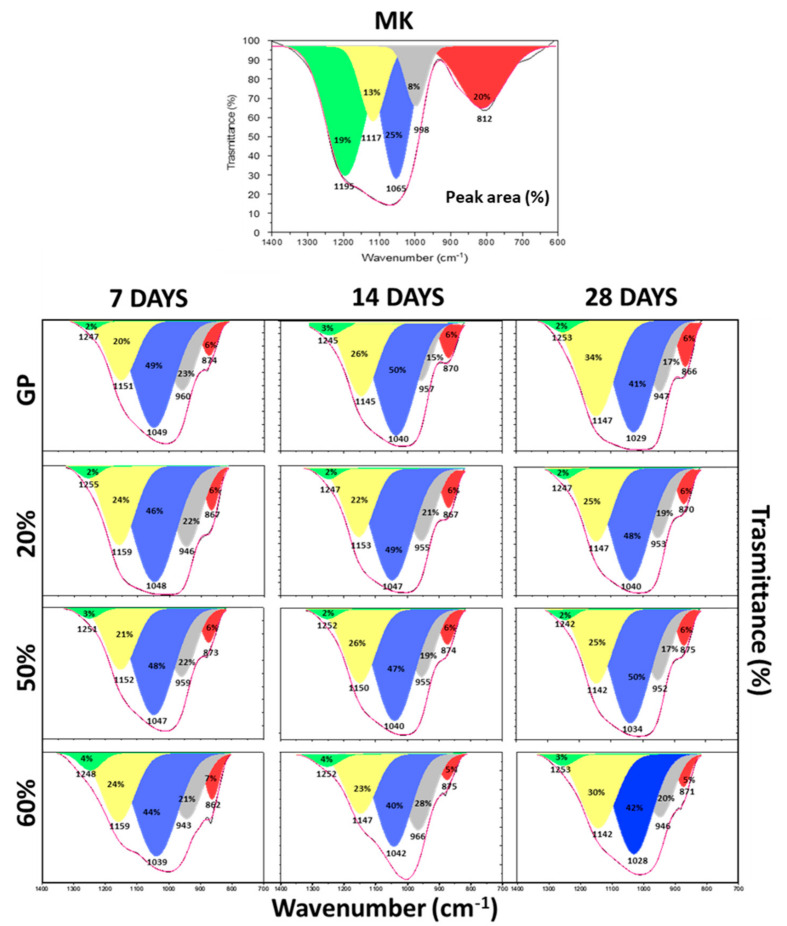
Deconvoluted DOSPM of FT-IR spectra of MK, GP, and GP/WG samples at different curing times. The percentages indicate the waste glass content.

**Figure 8:**
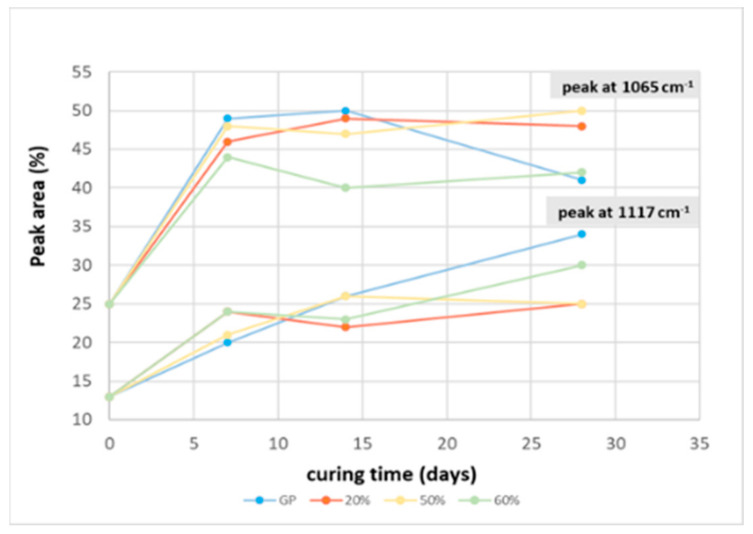
Comparison of the peak area determined from the deconvoluted DOSPM of FT-IR spectra of the two main peaks (1065 and 1117 cm^−1^) for MK (taken as reference value on *y*-axis), GP, GP/WG (20, 50 and 60%) samples at different curing times.

To better understand the evolution of the geopolymer 3D structures, FT-IR spectra were recorded at 7, 14, and 28 days of curing time (data not shown), and, as shown in Figure 7, all the DOSPM of GP and GP/WG (20, 50 and 60%) were deconvoluted (see Methods section for the deconvolution procedure), characterized, and compared to the MK DOSPM deconvolution. The MK deconvoluted spectrum was characterized by the strong band at 1065 cm^−1^, associated with also the bands at 1195 and 1117 cm^−1^, due to the asymmetric stretching of Si-O-T [19]. The band at 998 cm^−1^ can be assigned to Si-O of incompletely calcined metakaolin [19,31], while the band at 812 cm^−1^ can be ascribed to O-Al-O bending vibration of AlO_4_ tetrahedra [19,32]. After the alkali activation of metakaolin, following the geopolymerization at different curing times similar to MK derived GP, in all the spectra with an increased amount of WG, the intensity of the Si-O-T stretching bands changed gradually over time, indicating MK dissolution via hydrolysis reaction. These changes were also due to the Al inclusion, reorganization, and condensation of the gel structure. In accordance with [21], the appearance of the band at 1040–1020 cm^−1^ could be related to a silicate network with a lower extent of Al substitution. Looking at the blue band (1065 cm^−1^), the yellow band (1117 cm^−1^), and the green one (1195 cm^−1^) in the MK spectrum, one can notice that the first band shifts to a lower wavenumber and possesses an increased area (from 25% to 40–50%) in all the geopolymer products (during the curing time), while the second and the third bands are both characterized by a shift at higher wavenumbers, but possess different areas in all the samples. Moreover, the green band shows a strong area reduction (from 19% to 2–3%), while, on the contrary, the yellow one reveals an increased area (from 13% to 20–34%) of the whole deconvoluted range. During the geopolymerization the MK grey band at 998 cm^−1^ disappears and a new band detected at 960–943 cm^−1^ appears in the geopolymer samples. This band can be assigned to asymmetric stretching of non-bridging Si-O-Na type structure [18,19] and its contribution is very high in all the GP/WG samples (15–28% of the total area). Finally, the MK red band (812 cm^−1^) vanishes and a new band emerges at 874–862 cm^−1^ due to the -OH bending of Si-OH groups [19], whose existence negatively affects the degree of condensation [13]. Despite that, the presence of Si-OH groups is very low as the areas of red bands (5–6%) are not significant with respect to the other band contributions. Regarding all the deconvoluted spectra, Figure 8 shows also that GP and GP/WG 20 and 50% are almost geopolymerized after 7 days of curing time, while the structure of the sample with 60% of WG content is in constant evolution, stabilizing only at 28 days of curing time.

### 3.5. Thermogravimetric Analysis

**Figure 9:**
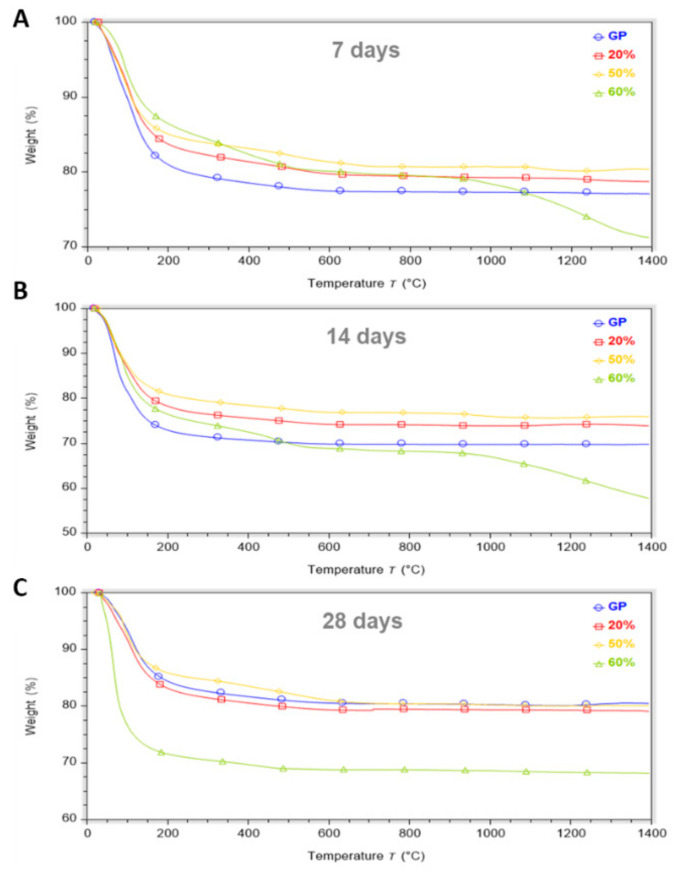
TGA of GP, GP/WG samples for 7 (**A**), 14 (**B**), 28 (**C**) days of curing time.

TGA was used to evaluate the thermal stability of metakaolin-based geopolymers after replacing MK with different percentages of waste glass powder. The thermal behaviour for GP and GP/WG at different curing times (7, 14, 28 days) are shown in Figure 9.

All GP and GP/WG samples show a first sharp weight loss between 25–200 °C associated with the evaporation of free and chemical bond water, but we noticed that GP retained more water with respect to the GP/WG samples at 7 (Figure 9A) and 14 (Figure 9B) days of curing time [10]. The explanation for this behavior is found in the increasing amount of glass particles that form compact bodies preventing the formation of pores in geopolymer structure [33,34]. The mass loss in the range of 200–500 °C is associated with the dihydroxylation of the Si-OH and Al-OH groups presented in the geopolymers [35].

All samples can be considered thermostable starting from about 600 °C with a total weight loss of 20–30%. An exception is GP/WG 60%, which has a further weight loss at a temperature higher than 900 °C of 8–11%, due to the decarbonization of carbonates by-products such as CaCO_3_ and Na_2_CO_3_ formed due to an excess of unreacted NaOH and CO_2_ (Figure 9A,B) [36]. This event is not visible for GP/WG 60% after 28 days of curing time (Figure 9C).

For 28 day of curing time, all TGA curves, except for GP/WG 60%, show the same mass loss of about 20%. This suggests a stabilization of thermal behaviors for GP at 20% and 50% for this curing time, while the results at 60% still show an evolving geopolymeric structure.

As Figure 9 illustrates (A,B,C), when observing TGA curves when the amount of curing time changes, compared to 7 and 28 day samples, a greater water loss for samples at 14 days (GP, 20% and 50%) is shown.

**Figure 10:**
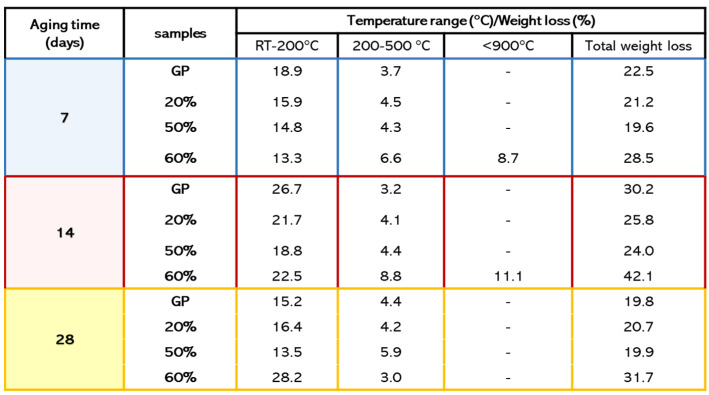
TGA data summary of the samples extracted after different curing times.

For Hajimohammadi et al. 2018 [4], the increase of weight loss versus curing time was an indication of a higher amount of geopolymer gel formation over time. In fact, when the development of geopolymer gel occurs, water is released back to the system and consequently, more trapped unbound water is collected within the pores. This can explain why we observe an increased weight loss for samples after 14 days of curing time (GP, 20 and 50%) with respect to samples after 7 days (Figure 10) [4].

Moreover, by altering the results for samples at 14 days of curing time, geopolymers can absorb a large amount of water, such as some of the moisture from the air after their extraction but prior to TGA testing [36].

In the samples cured for 28 days, we observed a decrease of weight loss (water content) in GP, and in the formulations with 20 and 50 wt % of WG. In the sample with 60 wt % of WG, the reactivity is still going on, thus liberating liquid water from the polycondensation reaction of the geopolymer aluminosilicate network, confirming the delay already observed in the FT-IR analyses as well in extant literature [14].

### 3.6. Antibacterial Activity 

**Figure 11:**
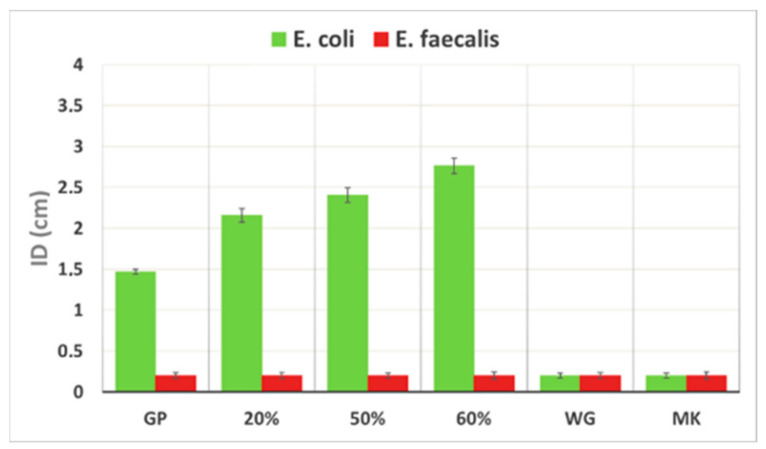
Comparison of inhibition halo of *E. coli* and *E. faecalis* for MK, WG, GP, and GP/WG samples. The percentages indicate the waste glass content in the geopolymer formulations. Please consider that the dimension of the IDs is in relation to Petri Plate diameter (PPD) (6 cm), thus the maximum value for ID is 6 cm.

Figure 11 shows the inhibition halos for *E. coli* and *E. faecalis* in presence of MK, WG (as precursors), GP, and GP/WG samples after 28 days of curing time. MK and WG have no antibacterial properties on both the bacterial strains, while the GP and GP/WG samples possess antibacterial properties concerning *E. coli*. The data also reveal that an increase of WG content corresponds to an increase of the ID dimension on *E. coli*, suggesting an improved ability to inhibit bacterial growth. Regarding the sample’s effect on *E. faecalis*, there is no evidence of antibacterial properties. The obtained data are in accordance with the 2018 study by Rubio-Avalos [37], which compared the antibacterial activity of metakaolin-based geopolymers treated, or not, with “triclosan” against *E. coli* (as gram negative) and *S. aureus* (as gram positive). In conclusion, after 28 days of curing time, the synthesized samples have antibacterial properties on the gram-negative bacteria assayed, and no influence on the gram-positive bacteria used in this study.

## 4. Conclusions

Waste glass favors the minimization of high pollution in cement production when added as a fine aggregate in place of river sand or other types of natural sand. In our geopolymer formulations, amounts of 20 to 60 wt % of ground container glass with a mix grain size proved to act as a reactive silica source [8,9], as well as a fine aggregate.

Waste glass provides similar reaction products to metakaolin in geopolymers, yet a delay has been noticed in the FT-IR vibrational spectra as well as in the TGA curves for the sample with 60 wt % of WG.

Even though we did not pre-wash the crushed WG before dry grinding, the final consolidated geopolymers do not have a strong impact on living organisms, showing a negative effect only on *E. coli* (gram-negative) and no effect on *E. faecalis* (gram-positive).

We can propose our compositions for building materials or surface finishing when a high WG content is used. In the case of coating, our formulation can provide an antibacterial surface for specific colonies of microorganisms.

## Data Availability

The data presented in this study are available on request from the corresponding author.
